# Development and validation of a population-based prediction model for prevalent hypertension: evidence from Qatar biobank

**DOI:** 10.3389/fcvm.2026.1866507

**Published:** 2026-09-04

**Authors:** Mustapha Mohammed, Ahmed Malki

**Affiliations:** 1 Biomedical Research Center, QU Health, Qatar University, Doha, Qatar; 2 Department of Biomedical Sciences, College of Health Sciences, QU Health, Qatar University, Doha, Qatar

**Keywords:** bootstrap resampling, calibration, diagnostic prediction model, discrimination, prevalent hypertension, Qatar biobank

## Abstract

**Background:**

Hypertension is a major cause of cardiovascular morbidity and mortality globally. Despite its high burden, validated population-based models and scores for prevalent hypertension remain limited. This study aimed to develop and internally validate a diagnostic model and scores to predict prevalent hypertension among adults in Qatar.

**Methods:**

This population-based study included 6,927 adults aged ≥18 years with complete baseline data for hypertension status from the Qatar Biobank (QBB). A multivariable logistic regression model was developed using a randomly selected training cohort (70%; *n* = 4,835) and internally validated in an independent testing cohort (30%; *n* = 2,092). Model performance was evaluated for discrimination using the area under the receiver operating characteristic curve (AUC), calibration using calibration plots and the Hosmer–Lemeshow goodness-of-fit test, and stability via bootstrap resampling (1,000 replications). Regression coefficients were converted into simplified point-based scores to facilitate practical application.

**Results:**

Overall, 1,097 (15.8%) participants had prevalent hypertension. The final model included predictors such as age, gender, nationality, body mass index, smoking, physical activity, sleep duration, diabetes, hyperlipidemia, and cardiovascular diseases. The model demonstrated good discrimination, with AUCs of 0.803 (95% CI: 0.78–0.83) and 0.797 (95% CI: 0.76–0.84) in the training and testing cohorts, respectively. Calibration was satisfactory, with close agreement between predicted and observed outcomes and non-significant Hosmer–Lemeshow tests in both the training (*p* = 0.102) and testing (*p* = 0.393) cohorts. Bootstrap bias across all predictors was negligible, further supporting the model's stability. A simplified 100-point predictive score was derived from the final model to support practical classification of hypertension.

**Conclusion:**

We developed and internally validated a population-based prediction model and simplified predictive score for prevalent hypertension in Qatar. The model demonstrated good discrimination, calibration, and stability. The model may support population-level hypertension stratification, targeted screening, and complication-preventive interventions.

## Introduction

Hypertension is one of the leading modifiable risk factors for cardiovascular disease (CVD), accounting for substantial morbidity, mortality, and healthcare burden globally (1–3). More than 1.28 billion adults are estimated to have hypertension, yet many remain undiagnosed, untreated, or inadequately controlled (4, 5). Because hypertension is frequently asymptomatic, early identification of affected individuals is essential for preventing cardiovascular complications and reducing disease burden.

The burden of hypertension has increased considerably in the Middle East and North Africa (MENA), particularly the Gulf Cooperation Council (GCC) region, driven by rapid urbanization, population aging, and lifestyle changes, including physical inactivity, unhealthy dietary habits, and increasing obesity (4, 6). Despite the remarkable socioeconomic and demographic transitions over the past decades in Qatar, the country is challenged by the rising prevalence of hypertension and other cardiometabolic disorders, particularly diabetes, dyslipidemia, and obesity (7–11). These conditions often coexist and substantially increase the risk of cardiovascular events, emphasizing the need for effective population-based strategies to identify individuals with hypertension and other cardiovascular conditions and facilitate timely intervention.

Current hypertension detection relies primarily on opportunistic blood pressure measurement and clinical diagnosis (12, 13). Although these approaches remain the cornerstone of diagnosis, they may fail to identify individuals with unrecognized hypertension who have limited contact with healthcare services. Prediction models provide a complementary approach by combining multiple routinely available characteristics to estimate an individual's probability of having a disease, thereby supporting risk stratification and screening (13–15). Compared with complex machine learning algorithms, multivariable regression models offer greater transparency, interpretability, and ease of implementation in routine clinical practice and public health settings.

Several hypertension prediction models have been developed in North American, European, and Asian populations, including the Framingham Hypertension Risk Score and other multivariable models incorporating demographic, anthropometric, lifestyle, and metabolic factors (14, 16, 17). Similarly, other models have been derived in clinical and non-Arab settings (18–24). While these models generally demonstrate acceptable predictive performance, their applicability to the GCC region remains uncertain due to differences in genetic background, environmental exposures, lifestyle behaviors, and the high prevalence of cardiometabolic diseases. Consequently, locally developed and validated population-based prediction models could improve risk estimations, especially in non-clinical settings.

In Qatar, previous studies have primarily examined the epidemiology and risk factors of hypertension rather than developing clinically interpretable prediction models (9, 10, 25). Although a recent study developed machine learning models for hypertension classification using Qatar Biobank (QBB) data (26), the primary objective of the study was to optimize predictive performance using computational algorithms. Machine learning approaches often provide limited interpretability, require computational infrastructure, and have inherent limitations in translation. However, multivariable regression models provide transparent effect estimates and can be translated into simple point-based scoring systems suitable for bedside or community use.

Therefore, the present study aimed to develop and internally validate a diagnostic prediction model of prevalent hypertension and derive a simplified point-based score using routinely available predictors from the Qatar Biobank.

## Methods

### Study design

This was a population-based cross-sectional prediction modeling using data from the Qatar Biobank (QBB). The study was set to develop and internally validate a diagnostic prediction model for prevalent hypertension among adults in Qatar. The study was reported in accordance with the Strengthening the Reporting of Observational Studies in Epidemiology (STROBE) guideline for cross-sectional studies (27, 28) and the Transparent Reporting of a Multivariable Prediction Model for Individual Prognosis or Diagnosis (TRIPOD) statement (13).

### Study setting

Data were obtained from QBB, a large population-based cohort established in 2012 under the Qatar Precision Health Institute (QPHI) to recruit 60,000 participants. QBB enrolls Qatari nationals and long-term residents (≥15 years of residence in Qatar) aged ≥18 years and collects standardized demographic, lifestyle, anthropometric, clinical, laboratory, and biological data at baseline, with follow-up assessments every five years. Data collection is performed by trained personnel using standardized questionnaires, calibrated equipment, and uniform clinical protocols (11, 29, 30).

### Study population

The source population comprised QBB participants aged ≥18 years with available baseline information on hypertension status and candidate predictors. The data for the current study were collected from August to December 2025. Participants with missing hypertension outcome data or missing predictor data (>20%) were excluded from the final analysis.

### Study variables

#### Outcome

The outcome of this study was prevalent hypertension at baseline. Hypertension was defined according to the QBB protocol as meeting at least one of the following criteria: (i) systolic blood pressure ≥140 mmHg and/or diastolic blood pressure ≥90 mmHg measured during the clinical assessment; (ii) self-reported physician diagnosis of hypertension; or (iii) current use of antihypertensive medication (30). Blood pressure was measured by trained staff using calibrated automated devices following standardized procedures.

#### Predictors

Candidate predictors were prespecified based on epidemiological evidence, clinical relevance, and availability within the QBB dataset (10, 11, 26). Consistent with a prediction-focused modeling approach and TRIPOD recommendations, all prespecified predictors were retained in the final model irrespective of statistical significance (13, 31). All predictors were measured at recruitment using standardized QBB instruments, clinical assessments, and questionnaires.

The candidate predictors included: (i) sociodemographic variables: age in years, gender (male or female), and nationality (Qatari or non-Qatari); (ii) anthropometric measure: body mass index (BMI, kg/m^2^); (iii) lifestyle factors: smoking status (no or yes), physical activity (adequate vs. inadequate), and sleep duration (adequate vs. inadequate); and (iv) clinical comorbidities (no or yes): diabetes, hyperlipidemia, and cardiovascular disease. Cardiovascular diseases were represented by a composite variable that included history of heart attack, angina, or stroke.

Physical activity was assessed using the International Physical Activity Questionnaire (IPAQ) and categorized as active or inactive according to standardized metabolic equivalent (MET)-minute thresholds (32–34). Sleep duration was self-reported and categorized as adequate (≥7 h/night) or inadequate (<7 h/night) according to current adult sleep recommendations (35). All predictors were measured at baseline using standardized questionnaires and clinical assessments.

### Sample size

The study size was determined by the number of eligible QBB participants with complete data. The final analytical sample included 6,927 participants, of whom 1,097 had prevalent hypertension. Sample size adequacy was guided by the events-per-variable (EPV) principle for prediction modeling, which recommends at least 10 outcome events per candidate predictor to minimize overfitting and ensure stable coefficient estimates (13, 36). The final model included 10 prespecified predictors and required a minimum of 100 hypertension events. The training cohort contained 770 hypertension cases, substantially exceeding this recommendation.

#### Model development and validation

The dataset was randomly divided into a training cohort (70%; *n* = 4,835) for model development and a testing cohort (30%; *n* = 2,092) for internal validation. The random split preserved similar distributions of hypertension status between cohorts while enabling evaluation of model performance in an independent subset of participants (37, 38).

A multivariable logistic regression model was fitted in the training cohort by simultaneously entering all prespecified predictors. Predictor selection was based on prior evidence and clinical relevance rather than statistical significance, and no stepwise selection procedures were used because they may reduce model stability and predictive performance (39). The regression coefficients estimated in the training cohort were subsequently applied unchanged to the testing cohort for internal validation.

### Model performance

Model performance was evaluated in both the training and testing cohorts by assessing discrimination and calibration. Discrimination was quantified using the area under the receiver operating characteristic curve (AUC) with 95% confidence intervals (CI). Calibration was assessed using calibration plots comparing observed and predicted hypertension probabilities across deciles of predicted risk and the Hosmer–Lemeshow goodness-of-fit test (13). In addition, the robustness of the final multivariable logistic regression model was assessed using bootstrap resampling (1,000 replications). Bootstrap analysis evaluated the stability of the regression coefficients by estimating bootstrap standard errors, bias, and bias-corrected and accelerated (BCa) 95% CI, which were compared with the base model estimates to assess parameter stability and potential overfitting.

#### Point-based predictive score

To enhance interpretability and potential public health application, the final logistic regression model was converted into a simplified 100-point risk score using a coefficient-based scoring approach adapted from established cardiovascular risk score methodologies. Regression coefficients were rescaled and converted into integer points proportional to their relative contribution to the model. Age was categorized into 5-year intervals (range: 18–84 years), and BMI into 1 kg/m^2^ increments (range: 18.5−61.2 kg/m^2^), whereas binary predictors were assigned integer points based on their relative regression coefficients.

### Statistical analysis

Statistical analyses were conducted using IBM SPSS Statistics version 28. Missing data were assessed for hypertension status and candidate predictors prior to analysis. Given the limited extent of predictor missingness, no imputation was performed. Continuous variables are presented as mean ± standard deviation (SD), whereas categorical variables are presented as frequencies and percentages. Baseline characteristics were compared between participants with and without hypertension and between training and testing cohorts using independent-samples t-tests or Mann–Whitney U tests, as appropriate, while categorical variables were compared using chi-square or Fisher's exact tests. Multivariable logistic regression analyses, with and without bootstrapping, were conducted using the available data. Statistical significance was not used for predictor selection, consistent with TRIPOD recommendations (13). All statistical tests were two-sided, and *p* ≤ 0.05 was considered statistically significant.

#### Ethical considerations

The study used de-identified secondary data from the Qatar Biobank. Ethical approval was obtained from the Qatar Biobank Institutional Review Board, Qatar Precision Health Institute (Reference: E-2025-QPHI-RES-ACC-00381-0379). The Qatar University Institutional Review Board determined that the study was exempt from full review under the Qatar Ministry of Public Health regulations (Reference: QU-IRB 318/2025-EM). All QBB participants provided informed consent for the use of their data and biological samples for approved research, and no identifiable participant information was accessed during this study.

## Results

### Study participants' screening and selection

**Figure 1:**
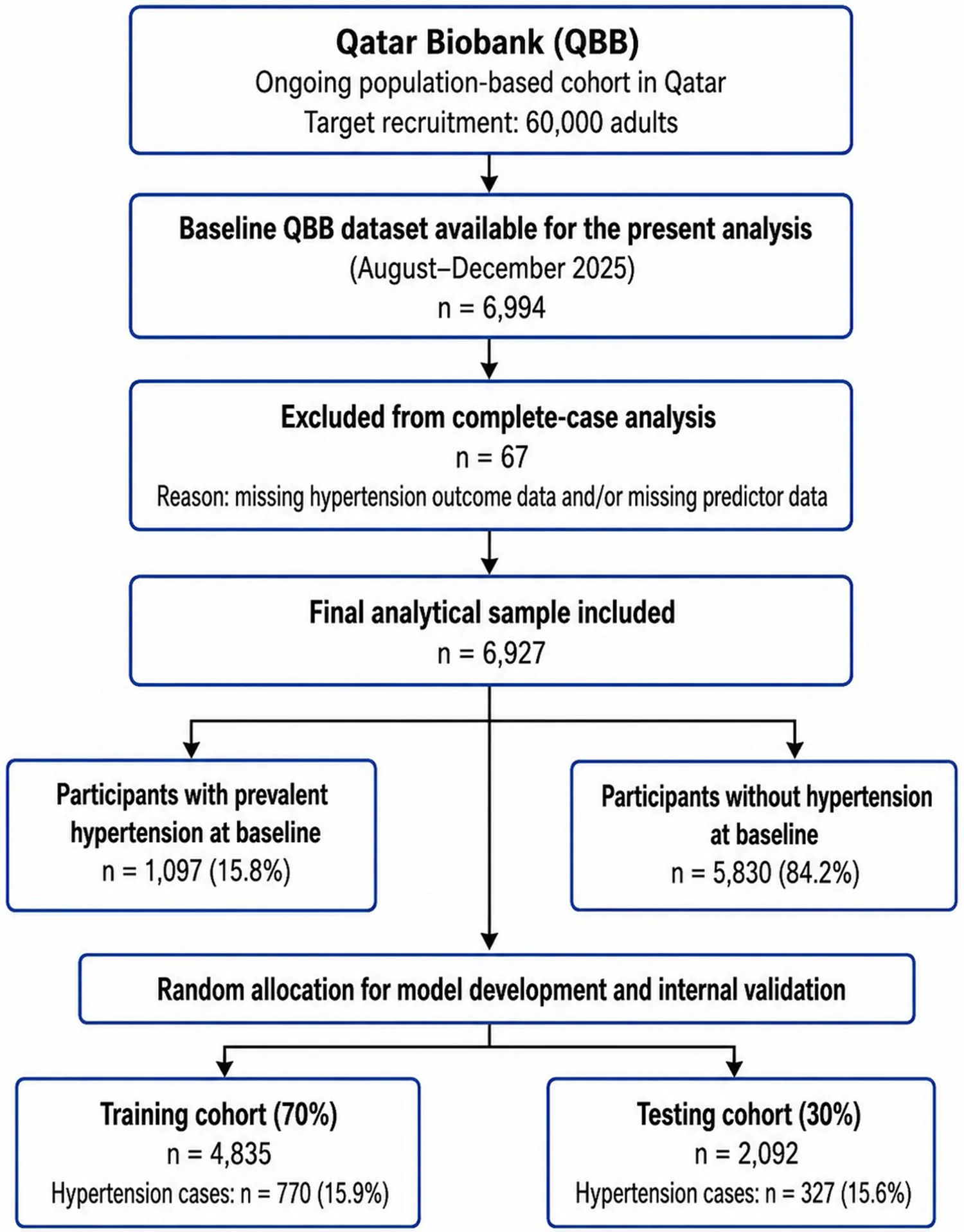
Flowchart of participants' selection for inclusion in the study.

A total of 6,994 participants' datasets were available from our study search query in the QBB portal during the data collection period. Sixty-seven were excluded due to missing hypertension outcome data and/or predictor (≥20%) data, resulting in a final analytical sample of 6,927 participants. Among these, 1,097 (15.8%) had prevalent hypertension and 5,830 (84.2%) did not have hypertension at baseline. The final sample was randomly allocated into a training cohort comprising 4,835 participants (70%), including 770 hypertension cases (15.9%), and a testing cohort comprising 2,092 participants (30%), including 327 hypertension cases (15.6%), for model development and internal validation, respectively (Figure 1).

### Baseline characteristics of the study population

A total of 6,927 participants were included in the final analysis, of whom 1,097 (15.8%) had prevalent hypertension. The overall mean age was 41.4 ± 12.1 years, 64.3% were male, 69.8% were Qatari nationals, and the mean BMI was 29.5 ± 5.7 kg/m^2^. Overall, 64.4% of participants reported inadequate sleep duration. Among participants with hypertension, the mean age was 48.8 ± 12.1 years, 63.8% were male, 67.9% were Qataris, and the mean BMI was 32.3 ± 5.9 kg/m^2^. Diabetes, hyperlipidemia, and cardiovascular disease were more prevalent among participants with hypertension than among those without hypertension (Table 1).

**Table 1:** Baseline characteristics of the study population.

Variables	Category	Hypertension, *n* (%)		*Total, *n* (%)
		Yes (*N* = 1,097)	No (*N* = 5,830)	*N* = 6,927
Age (years)	Mean ± SD	48.8 ± 12.1	39.5 ± 11.4	41.4 ± 12.1
Gender	Female	397 (36.2)	2,074 (35.6)	2,471 (35.7)
	Male	700 (63.8)	3,756 (64.4)	4,456 (64.3)
Nationality	Qatari	745 (67.9)	4,089 (70.1)	4,834 (69.8)
	Non-Qatari	352 (32.1)	1,741 (29.9)	2,093 (30.2)
BMI (kg/m^2^)	Mean ± SD	32.3 ± 5.9	29.0 ± 5.6	29.5 ± 5.7
Physical activity	Inactive	232 (39.0)	996 (29.1)	1,228 (30.5)
	Active	363 (61.0)	2,430 (70.9)	2,793 (69.5)
Sleeping duration	Inadequate	750 (68.4)	3,709 (63.7)	4,459 (64.4)
	Adequate	347 (31.6)	2,113 (36.3)	2,460 (35.6)
Smoking status	Yes	360 (34.3)	1,979 (35.5)	2,339 (35.3)
	No	689 (65.7)	3,591 (64.5)	4,280 (64.7)
Diabetes	Yes	461 (42.1)	666 (11.4)	1,127 (16.3)
	No	635 (57.9)	5,159 (88.6)	5,794 (83.7)
Hyperlipidemia	Yes	668 (61.5)	1,546 (26.8)	2,214 (32.3)
	No	419 (38.5)	4,222 (73.2)	4,641 (67.7)
CVD status	Yes	26 (2.4)	37 (0.6)	63 (0.9)
	No	1,061 (97.6)	5,731 (99.4)	6,792 (99.1)

BMI, body mass index; CVD, cardiovascular diseases (composite of heart attack, angina, and stroke); SD, standard deviation; *N = 6,927 participants with complete hypertension outcome data, while variable-specific totals may differ because of missing values in some predictor variables.

### Comparisons of the characteristics of the study cohorts

The dataset was randomly divided into a training cohort comprising 4,835 participants (70%) and a testing cohort comprising 2,092 participants (30%) (Figure 1). The prevalence of hypertension was comparable between the training (15.9%) and testing (15.6%) cohorts. Likewise, demographic, lifestyle, anthropometric, and clinical characteristics were similar between the two cohorts, with no statistically significant differences observed (*p* > 0.05), indicating that the random split produced well-balanced development and validation datasets (Table 2).

**Table 2:** Comparisons of the characteristics of the study cohorts.

Variables	Category	Hypertension Status						*p*-value
		Training Cohort			Testing Cohort			
		Yes (*N* = 770)	No (*N* = 4,065)	Total (*N* = 4,835)	Yes (*N* = 327)	No (*N* = 1,765)	Total (*N* = 2,092)	
Age (years)	Mean ± SD	49.7 ± 11.4	40.0 ± 11.6	41.9 ± 12.0	48.8 ± 12.1	39.5 ± 11.4	41.6 ± 12.2	0.345
BMI (kg/m^2^)	Mean ± SD	32.1 ± 5.9	28.9 ± 5.6	29.7 ± 5.8	32.3 ± 5.9	29.0 ± 5.6	29.8 ± 5.9	0.515
Gender	Female	291 (37.8)	1,454 (35.8)	1,745 (36.1)	106 (32.4)	620 (35.1)	726 (34.7)	0.280
	Male	479 (62.2)	2,611 (64.2)	3,090 (63.9)	221 (67.6)	1,145 (64.9)	1,366 (65.3)	
Nationality	Qatari	526 (68.3)	2,849 (70.1)	3,375 (69.8)	219 (67.0)	1,240 (70.3)	1,459 (69.7)	0.982
	Non-Qatari	244 (31.7)	1,216 (29.9)	1,460 (30.2)	108 (33.0)	525 (29.7)	633 (30.3)	
Physical activity	Inactive	180 (41.8)	708 (29.3)	888 (31.2)	52 (31.7)	288 (28.5)	340 (28.9)	0.167
	Active	251 (58.2)	1,707 (70.7)	1,958 (68.8)	112 (68.3)	723 (71.5)	835 (71.1)	
Sleeping	Inadequate	523 (67.9)	2,605 (64.2)	3,128 (64.8)	227 (69.4)	1,104 (62.7)	1,331 (63.7)	0.419
	Adequate	247 (32.1)	1,455 (35.8)	1,702 (35.2)	100 (30.6)	658 (37.3)	758 (36.3)	
Smoking	Yes	237 (32.5)	1,377 (35.5)	1,614 (35.0)	123 (38.6)	602 (35.7)	725 (36.1)	0.382
	No	493 (67.5)	2,506 (64.5)	2,999 (65.0)	196 (61.4)	1,085 (64.3)	1,281 (63.9)	
Diabetes	Yes	327 (42.5)	473 (11.6)	800 (16.6)	134 (41.0)	193 (10.9)	327 (15.6)	0.357
	No	442 (57.5)	3,588 (88.4)	4,030 (83.4)	193 (59.0)	1,571 (89.1)	1,764 (84.4)	
Hyperlipidemia	Yes	468 (61.3)	1,099 (27.3)	1,567 (32.7)	200 (61.7)	447 (25.6)	647 (31.3)	0.251
	No	295 (38.7)	2,925 (72.7)	3,220 (67.3)	124 (38.3)	1,297 (74.4)	1,421 (68.7)	
CVD status	Yes	18 (2.4)	22 (0.5)	40 (0.8)	8 (2.5)	15 (0.9)	23 (1.1)	0.335
	No	745 (97.6)	4,002 (99.5)	4,747 (99.2)	316 (97.5)	1,729 (99.1)	2,045 (98.9)	

BMI, body mass index; CVD, cardiovascular diseases (composite of heart attack, angina, and stroke); SD, standard deviation; *p* ≤ 0.05 was considered statistically significant using independent-samples t-tests for continuous variables and Chi-square (or Fisher's exact) tests for categorical variables.

### Development of the prediction model

A multivariable logistic regression model was developed in the training cohort using ten prespecified predictors selected based on clinical relevance and prior evidence. The final model included age, gender, nationality, BMI, smoking status, physical activity, sleep duration, diabetes, hyperlipidemia, and cardiovascular disease. Increasing age, BMI, diabetes, hyperlipidemia, and poor sleep were independently associated with prevalent hypertension, whereas gender, nationality, smoking, physical activity, and cardiovascular disease did not reach statistical significance after adjustment. Regression coefficients, adjusted odds ratios, 95% confidence intervals, and *p*-values are presented in Table 3.

**Table 3:** Multivariable prediction model for prevalent hypertension.

Variable	Category	β	SE	Wald	*p*-value	Exp(β)	95% CI
Age (years)	-	0.045	0.006	58.88	<0.001	1.05	1.03–1.06
BMI (kg/m^2^)	-	0.088	0.011	60.20	<0.001	1.09	1.07–1.12
Gender	Female	0.135	0.139	0.95	0.330	1.15	0.87–1.50
	Male	1					
Nationality	Qatari	0.065	0.129	0.26	0.614	1.07	0.83–1.38
	Non-Qatari	1					
Physical activity	Inactive	0.196	0.125	2.46	0.117	1.22	0.95–1.55
	Active	1					
Sleep duration	Inadequate	0.287	0.134	4.62	0.032	1.33	1.03–1.73
	Adequate	1					
Smoking	Yes	0.178	0.155	1.31	0.252	1.19	0.88–1.62
	No	1					
Diabetes	Yes	1.031	0.135	58.56	<0.001	2.80	2.15–3.65
	No	1					
Hyperlipidemia	Yes	0.847	0.126	45.19	<0.001	2.33	1.82–2.99
	No	1					
CVD status	Yes	0.581	0.474	1.50	0.221	1.79	0.71–4.30
	No	1					
Constant	-	−7.438	0.483	236.69	<0.001	0.001	−8.07–7.19

BMI, body mass index; CVD, cardiovascular diseases (composite of heart attack, angina, and stroke); SD, standard deviation; β, beta coefficient; SE, standard error; Exp(β), odds ratio; CI, confidence interval; *p* ≤ 0.05 was considered statistically significant using multivariable logistic regression.

### Prediction model validation and performance

**Figure 2:**
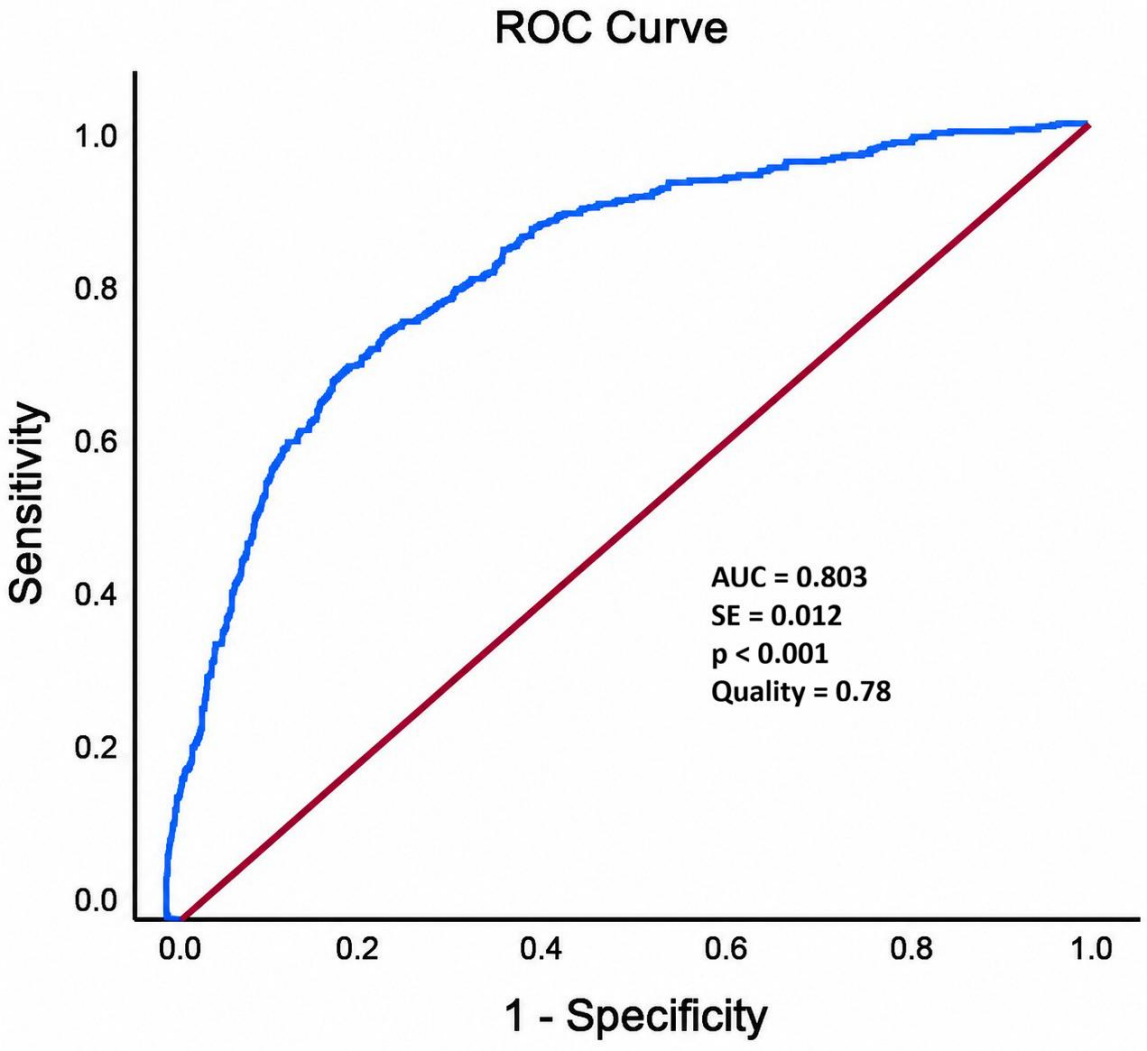
Discrimination ROC curve of the prevalent hypertension model in the training cohort.

**Figure 3:**
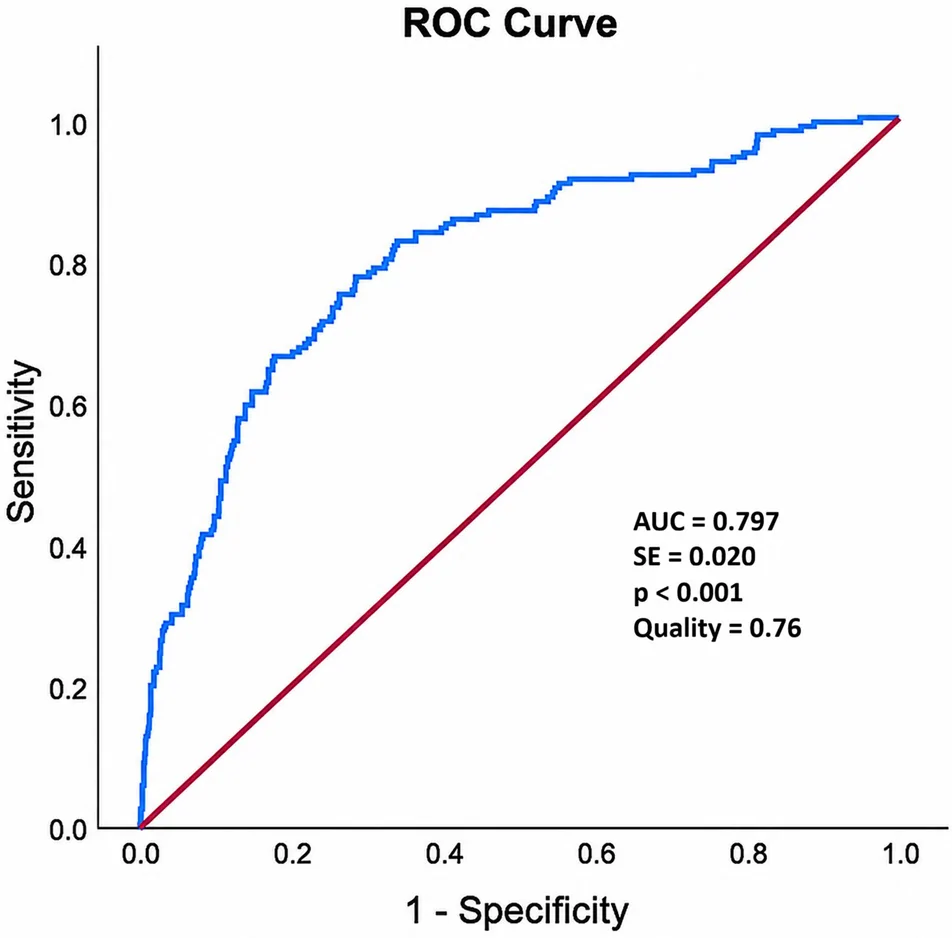
Discrimination ROC curve of the prevalent hypertension model in the testing cohort.

The model demonstrated good discrimination in both cohorts, with an AUC of 0.803 (95% CI: 0.78–0.83; *p* < 0.001) in the training cohort and 0.797 (95% CI: 0.76–0.84; *p* < 0.001) in the testing cohort. Overall model quality was comparable between the training (0.78) and testing (0.76) cohorts (Figures 2, 3).

**Figure 4:**
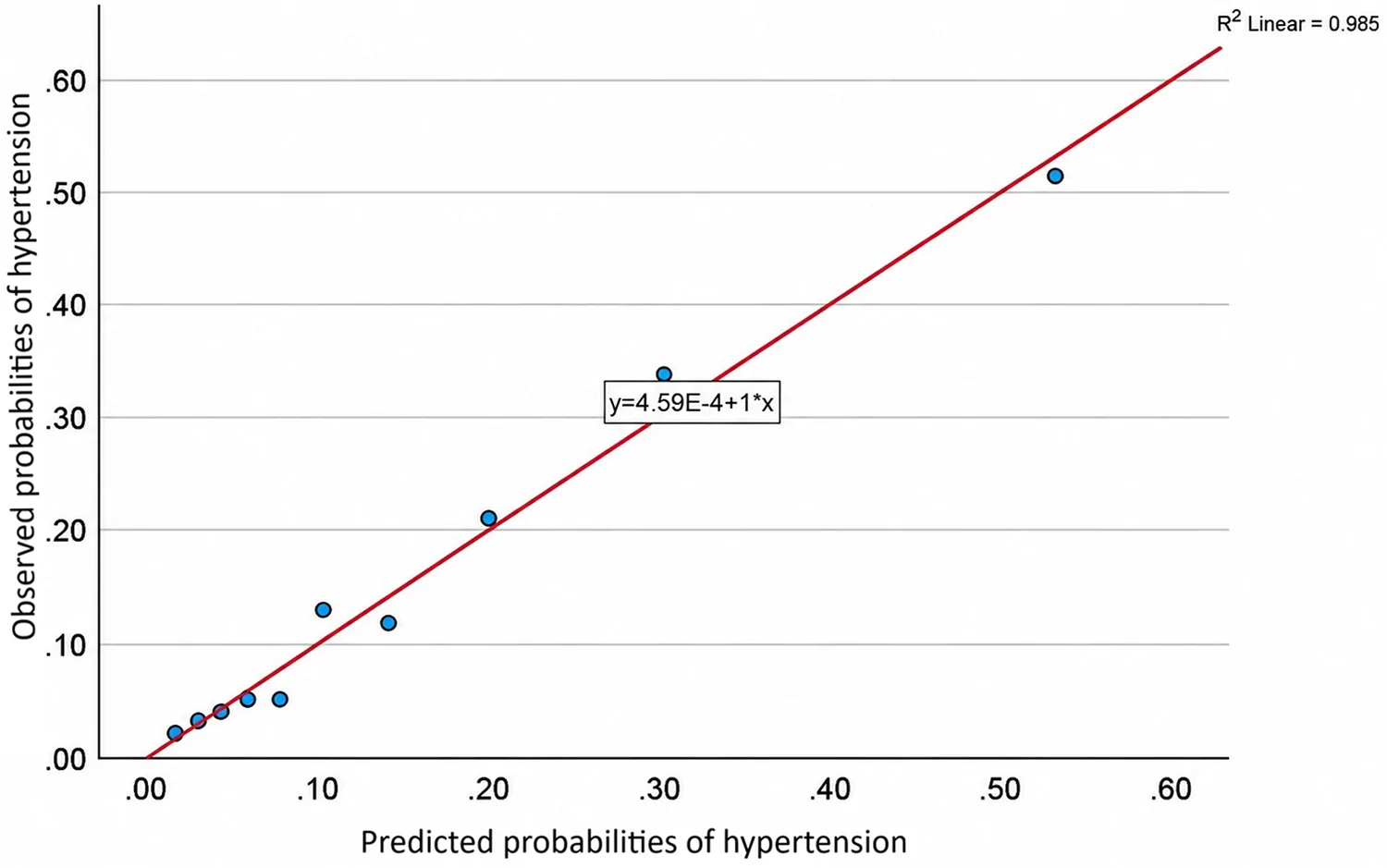
Calibration plot of prevalent hypertension model in the training cohort.

**Figure 5:**
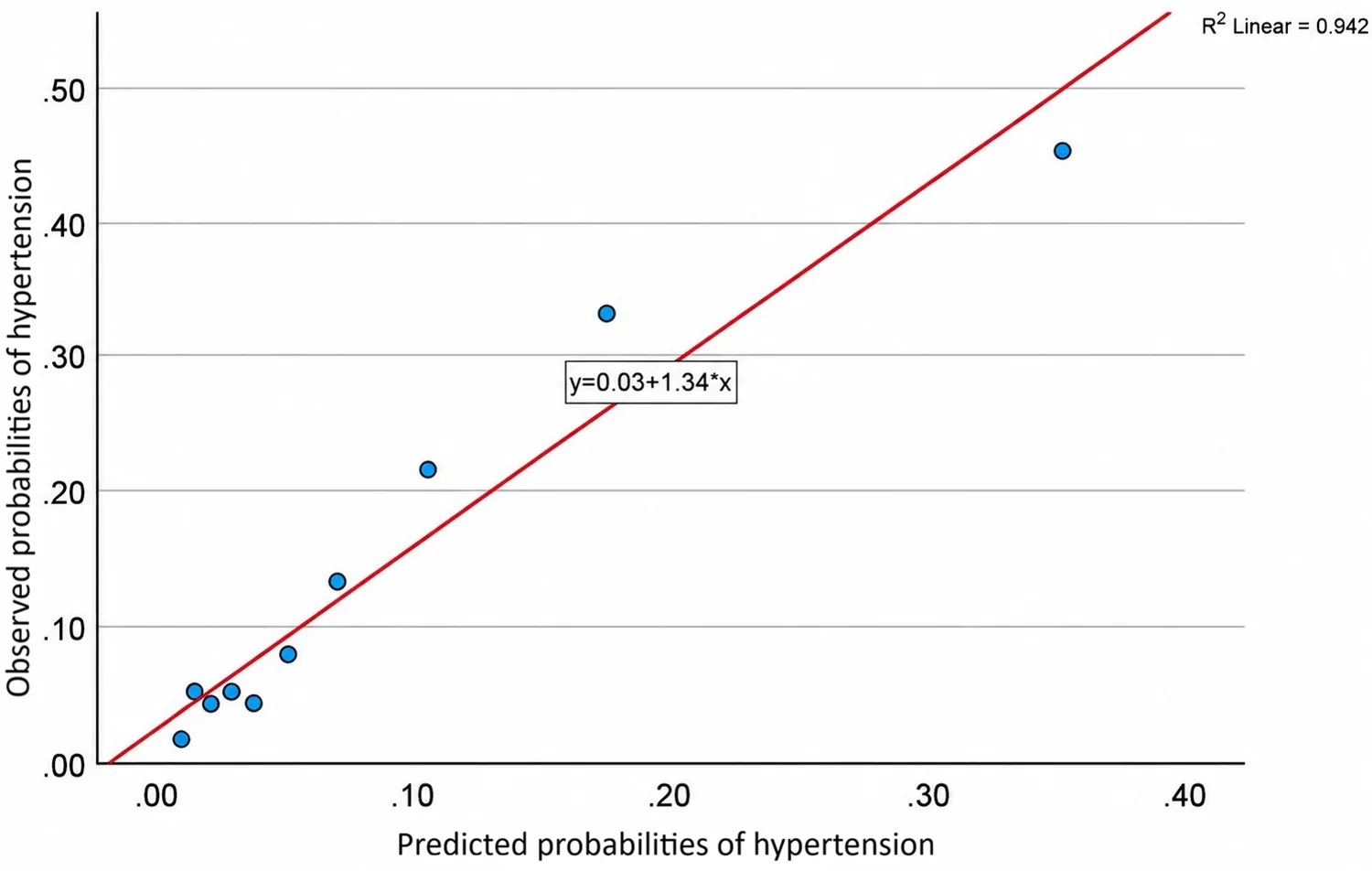
Calibration plot of prevalent hypertension model in the testing cohort.

Calibration analysis demonstrated close agreement between predicted and observed hypertension probabilities in both cohorts. The Hosmer–Lemeshow goodness-of-fit test indicated no evidence of poor fit in either the training (χ^2^ = 13.31; *p* = 0.102) or testing (χ^2^ = 8.42; *p* = 0.393) cohort (Figures 4, 5).

Moreover, the optimal probability threshold determined using Youden's Index was 0.157 (maximum Youden's Index = 0.474), yielding a sensitivity of 72.5%, specificity of 74.9%, positive predictive value (PPV) of 35.2%, and negative predictive value (NPV) of 93.5%.

### Bootstrap validation of the prediction model

Bootstrap validation using 1,000 resamples demonstrated good stability of the final multivariable logistic regression model (Table 4). The bootstrap regression coefficients were comparable to the base-model estimates, with minimal bias observed across all predictors. Age (β = 0.044, BCa 95% CI: 0.03–0.06), BMI (β = 0.097, BCa 95% CI: 0.08–0.12), sleep duration (β = 0.283, BCa 95% CI: 0.07–0.49), smoking (β = 0.266, BCa 95% CI: 0.01–0.51), diabetes (β = 1.050, BCa 95% CI: 0.82–1.27), and hyperlipidemia (β = 0.798, BCa 95% CI: 0.56–1.05) remained statistically significant. Gender, nationality, physical activity, and cardiovascular disease remained non-significant, consistent with the base model.

**Table 4:** Bootstrap validation of the prediction model of prevalent hypertension.

Variable	Category	β	SE	Bias	*p*-value	BCa 95% CI
Age (years)	-	0.044	0.005	<0.001	<0.001	0.03–0.06
BMI (kg/m^2^)	-	0.097	0.009	0.009	<0.001	0.08–0.12
Gender	Female	0.239	0.121	0.006	0.050	0.01–0.51
	Male	1				
Nationality	Qatari	0.089	0.109	0.005	0.414	−0.14–0.33
	Non-Qatari	1				
Physical activity	Inactive	0.101	0.110	0.002	0.358	−0.13–0.31
	Active	1				
Sleep duration	Inadequate	0.283	0.108	0.001	0.010	0.07–0.49
	Adequate	1				
Smoking	Yes	0.266	0.129	−0.004	0.033	0.01–0.51
	No	1				
Diabetes	Yes	1.050	0.120	−0.002	<0.001	0.82–1.27
	No	1				
Hyperlipidemia	Yes	0.798	0.109	0.012	<0.001	0.56–1.05
	No	1				
CVD status	Yes	0.516	0.411	−0.028	0.196	−0.30–1.22
	No	1				
Constant	-	−7.714	0.416	−0.057	<0.001	−8.47–7.09

BMI, body mass index; CVD, cardiovascular diseases (composite of heart attack, angina, and stroke); SD, standard deviation; β, beta coefficient; BCa, bias-corrected and accelerated; *p* ≤ 0.05 was considered statistically significant using bootstrapping multivariable logistic regression.

### Development of the point-based predictive score

The final multivariable logistic regression model was translated into a simplified 100-point prediction score to facilitate clinical application (Table 5). Age contributed up to 24 points, BMI up to 43 points, and the remaining predictors up to 33 points. Among the binary predictors, diabetes received the highest weighting (10 points), followed by hyperlipidemia (8 points), cardiovascular disease (6 points), inadequate sleep (3 points), physical inactivity (2 points), smoking (2 points), female sex (1 point), and Qatari nationality (1 point). The total score ranged from 0 to 100 points, with higher scores corresponding to a greater predicted probability of hypertension (Table 4).

**Table 5:** Point-based predictive scores for prevalent hypertension.

Predictor	Category	β	Points
Age (years) (range: 18–84 years)	Per 5-year above 18–24 years	0.045	2–24
BMI (kg/m^2^) (range: 18.5–61.2 kg/m^2^)	Per 1 kg/m^2^ above 18.5 kg/m^2^	0.088	1–43
Gender	Female	0.135	1
	Male	Ref	0
Nationality	Qatari	0.065	1
	Non-Qatari	Ref	0
Physical activity	Inactive	0.196	2
	Active	Ref	0
Sleep quality	Inadequate	0.287	3
	Adequate	Ref	0
Smoking	Yes	0.178	2
	No	Ref	0
Diabetes	Yes	1.031	10
	No	Ref	0
Hyperlipidemia	Yes	0.847	8
	No	Ref	0
CVD status	Yes	0.581	6
	No	Ref	0
Total score			0–100

BMI, body mass index; CVD, cardiovascular diseases (composite of heart attack, angina, and stroke); SD, standard deviation; β, beta coefficient; Ref, reference group; The total score ranges from 0 to 100 points, with higher scores indicating a greater predicted risk of prevalent hypertension.

Total score = Age (2) [per 5-year above 18–24 years] + BMI (1) [per 1 kg/m^2^ above 18.5 kg/m^2^] + Female (1) + Qatari (1) + Physically inactive (2) + Inadequate sleep (3) + Smoking (2) + Diabetes (10) + Hyperlipidemia (8) + Cardiovascular disease (6).

## Discussion

This population-based study developed and internally validated a predictive model to classify individuals with prevalent hypertension using data from the Qatar Biobank. The final model incorporated readily available demographic, lifestyle, and clinical predictors. The final predictive model demonstrated good discrimination and calibration across all the studied cohorts. In addition, the model was translated into a simplified 100-point risk score, providing a practical tool for estimating an individual's likelihood of being classified as hypertensive.

The high prevalence of hypertension and the associated risk factors, including diabetes, hyperlipidemia, and cardiovascular disease, demonstrate a considerable burden of chronic non-communicable diseases in Qatar. These findings are consistent with previous studies in Qatar and the broader MENA region, which have reported strong clustering of hypertension with cardiometabolic risk factors (11, 25, 40, 41). The coexistence of these conditions reflects shared underlying mechanisms, including insulin resistance, inflammation, and vascular dysfunction, and highlights the importance of integrated disease assessment approaches.

The predictors retained in the final model are consistent with the current understanding of the pathophysiology of hypertension. Age and BMI were among the strongest contributors, reflecting the cumulative effects of vascular aging and adiposity on blood pressure regulation. Advancing age is associated with progressive arterial stiffening and endothelial dysfunction, whereas excess adiposity contributes to hypertension through activation of the sympathetic nervous system, the renin–angiotensin–aldosterone system, insulin resistance, and chronic low-grade inflammation (42–44). These findings are consistent with previous hypertension prediction models, including the Framingham Hypertension Risk Score and other population-based models, in which age and measures of adiposity consistently emerge as dominant predictors (14, 16).

Diabetes and hyperlipidemia were also strong independent predictors of prevalent hypertension. Their prominent contribution reflects the well-established clustering of metabolic disorders, which share common biological pathways with hypertension, including endothelial dysfunction, oxidative stress, vascular inflammation, and insulin resistance (45). These conditions share overlapping biological pathways and frequently coexist in individuals, particularly in populations with high prevalence of obesity and sedentary lifestyles (46–49). In the context of Qatar, where diabetes and dyslipidemia are common at both population and clinical levels (49–52). Incorporating these conditions substantially improves the model's clinical relevance and supports an integrated cardiometabolic approach to risk assessment, rather than considering hypertension in isolation.

Sleep quality emerged as a significant lifestyle predictor of hypertension, while physical inactivity and smoking, although directionally associated, were not statistically significant. This finding adds to growing evidence that inadequate sleep contributes to blood pressure dysregulation through alterations in autonomic balance, hypothalamic–pituitary–adrenal axis activation, circadian rhythm disruption, and systemic inflammation (53). Although smoking and physical inactivity were not independently associated with hypertension after multivariable adjustment, both were retained because they established cardiovascular risk factors, improved the clinical interpretability of the model, and remained important targets for primary prevention.

The final model demonstrated good discriminative ability, with high AUC values in both the training and the testing cohort. These values are comparable to those reported in other hypertension prediction models, which typically show moderate-to-good discrimination (16, 19). The consistency of AUC values between the training and testing datasets suggests that the model is not substantially overfitted and performs reliably on unseen data, supporting its internal validity. Moreover, the calibration performance was also satisfactory, with close agreement between predicted and observed hypertension status. Good calibration is essential for practical application, as it ensures that predicted status corresponds to actual outcome frequencies (54). These findings are consistent with established principles in prediction modeling, which emphasize the importance of both discrimination and calibration in evaluating model performance (13, 54). To further evaluate model robustness, we additionally performed bootstrap resampling, which demonstrated stable regression coefficients with minimal bias, supporting the reliability of the final model.

Our study complements the previous Qatar Biobank study that evaluated multiple machine learning algorithms for hypertension prediction (26). The present study prioritized developing an easily interpretable and implementable prediction model. Unlike many machine learning approaches, logistic regression provides directly interpretable effect estimates, enables comprehensive assessment of both discrimination and calibration, and facilitates easier translation. Moreover, our model incorporated routinely available demographic, lifestyle, and clinical variables to improve its applicability in routine practice and public health settings, making it more suitable for community-based screening.

One additional strength of this study is the development of a simplified 100-point prediction score derived from the final regression model. The scoring system preserves the relative contribution of each predictor while eliminating the need for specialized computational tools. Diabetes, hyperlipidemia, and cardiovascular disease contributed the highest scores among the binary predictors, whereas age and BMI contributed progressively increasing points consistent with their continuous association with hypertension. Similar coefficient-based scoring systems have been widely adopted in cardiovascular medicine because they simplify risk estimation, maintain acceptable predictive performance, and facilitate clinical decision-making (55, 56).

However, some important limitations should be acknowledged. First, the analysis was based on cross-sectional baseline data, which enabled the model to predict prevalent rather than incident hypertension. Second, certain variables, such as physical activity, were self-reported and may be subject to recall bias. Third, although the model was internally validated with an additional stable bootstrap model to improve robustness, external validation in independent populations is necessary to assess its generalizability and transferability. Fourth, to minimize overfitting and comply with the recommended 10-EPV principle, predictor selection was restricted to a limited number of variables, and some potentially relevant risk factors (e.g., waist circumference, dietary factors, family history, and psychosocial variables) were not included.

## Conclusion

We developed and internally validated a multivariable prediction model and simplified point-based predictive score for prevalent hypertension using a large population-based cohort from the Qatar Biobank. The model demonstrated good discrimination, calibration, and stability, indicating its potential utility for population-based disease identification using routinely available demographic, lifestyle, and clinical information. The simplified point-based score enhances the model's interpretability and may facilitate population-level disease stratification, targeted screening, and early interventions for disease progression. Further external validation and prospective evaluation are warranted before clinical implementation.

## Data Availability

The data analyzed in this study is subject to the following licenses/restrictions: The dataset generated for this study is available on request to the QPHI-QBB. We are bound by the QPHI-QBB data and material transfer agreement to not further transfer data without the provider's prior written approval. Requests to access these datasets should be directed to Research Officer, QPHI-RO@qf.org.qa.
